# Molecular mechanisms of polysaccharide components of kidney-tonifying traditional Chinese medicine in regulating osteoporosis 

**DOI:** 10.3389/fphar.2026.1685724

**Published:** 2026-05-26

**Authors:** Zijing Zheng, Fan Yang, Xinxin Ye, Yingying Jin, Hanmin Zhu, Xi Zhou, Wenjing Zhang, Wei Li, Nan-Nan Shen

**Affiliations:** 1 School of Medicine, Shaoxing University, Shaoxing, Zhejiang, China; 2 School of Medicine, Stanford University, Stanford, CA, United States; 3 Department of Sports Science, Zhejiang University, Hangzhou, Zhejiang, China; 4 School of Clinical Medicine, Hubei University of Arts and Science, Xiangyang, China; 5 Department of Pharmacy, Affiliated Hospital of Shaoxing University, Shaoxing, Zhejiang, China

**Keywords:** osteoblast, osteoclast, osteoporosis, polysaccharide, tonification of the kidney, traditional Chinese medicine

## Abstract

Osteoporosis, a prevalent systemic skeletal disorder, poses a significant health risk to the elderly population, arising from a multitude of factors that disrupt bone homeostasis. This delicate balance is primarily regulated by the dynamic interplay between osteoblasts and osteoclasts. When this equilibrium is perturbed, osteoporosis can ensue. Traditional Chinese Medicine (TCM) posits that the kidney, as the repository of vital essence, is instrumental in the production of bone marrow, which in turn confers skeletal strength. Consequently, TCM therapies that focus on kidney replenishment are frequently employed in the management of osteoporosis and other bone-related conditions. The polysaccharide metabolites of these TCM, which are designed to invigorate the kidney, exhibit a spectrum of pharmacological activities and engage multiple targets, such as Wnt and BMP-2/Smads signaling pathway activation and oxidative stress reduction. Notably, the protective and therapeutic roles of these polysaccharides in osteoporosis are mediated through distinct mechanisms and pathways. This review provides an overview of the application of the polysaccharide metabolites of kidney-tonifying TCM in the field of osteoporosis, with special emphasis on the mechanisms underlying the pharmacological effects of the various polysaccharide metabolites. It meticulously examines the precise molecular underpinnings and pathways through which these polysaccharides influence osteogenesis and osteoclastogenesis, with the aim of providing a robust foundation for future investigative endeavors and clinical applications of these polysaccharide metabolites in TCM kidney-tonifying therapies. This review summarizes preclinical evidence from animal and cell studies. However, clinical translation remains limited as most findings await validation in human trials. Moreover, this review aims to bolster the empirical evidence supporting the TCM principle that “the kidney governs the bones,” thereby enriching the understanding and practical application of this ancient medical wisdom in contemporary healthcare settings.

## 1 Introduction

The National Health Commission’s 2020 data underscores osteoporosis as a predominant health issue for the demographic over 50 in China. Osteoporosis in middle-aged and elderly women exhibit an incidence rate roughly five times higher than their male counterparts. At the same time, a large number of individuals in China have low bone mass and belong to a high-risk group for osteoporosis; however, most Chinese have a poor understanding of osteoporosis.

The core pathogenesis of osteoporosis is an abnormal bone metabolism caused by various factors, which results in greater bone resorption than bone formation. Osteoblast differentiation originates from bone marrow mesenchymal stem cells (BMSCs). Furthermore, BMSCs can differentiate into chondrocytes and adipocytes, and the specific direction of differentiation depends on the regulation of molecular signals. Osteoblasts promote bone formation by promoting the synthesis, maturation, and mineralization of the bone matrix (Hu and Olsen, 2016). Differentiated bone-lining cells can protect bones from damage caused by osteoclast absorption and regulate bone formation in coordination with osteoclasts to maintain bone homeostasis (Ponzetti and Rucci, 2021).

Common drugs used for the treatment of osteoporosis include bisphosphonates, calcitonin, and parathyroid hormone analogs. They have good therapeutic effects when used alone or in combination; however, some drugs have noticeable side effects. Based on the theory of Traditional Chinese Medicine (TCM) that the kidneys govern the bones, many kidney-tonifying botanical drugs have been used to treat bone-related diseases (Wang F. et al., 2023). In “Huangdi Neijing”, there are many records of kidney tonification drugs used to treat osteoporosis directly or indirectly. Recent studies have also confirmed that TCM to tone the kidney can affect osteoblast and osteoclast activity and function through multiple signaling pathways and plays a role in the treatment of osteoporosis (Xu et al., 2019). In traditional Chinese medicine (TCM), the kidney system can regulate the neuroendocrine system by activating key brain regions or neurons in the hypothalamus, such as the arcuate nucleus or Proopiomelanocortin (POMC) neurons, thereby regulating the metabolic balance of osteoblasts and osteoclasts (Liang et al., 2025). It can also influence estrogen secretion through the hypothalamic-pituitary-gonadal axis, thereby regulating bone metabolism (Yao et al., 2025). The hypothalamic-pituitary-adrenal axis and the hypothalamic-pituitary-thyroid axis are also important pathways. The hormones they secrete can regulate bone metabolism by modulating the levels of calcium and phosphorus (Niwczyk et al., 2023). Polysaccharides are important metabolites of TCM for tonifying the kidney and are present in high concentrations in many TCMs (Zhang S. et al., 2024; Yang et al., 2024). Current studies have shown that polysaccharide metabolites of Chinese herbal medicines have various biological activities, including anti-diabetes, anti-virus, anti-tumor, anti-inflammatory, anti-oxidant, anti-radiation, lipid-lowering, and immune regulatory effects (Zeng et al., 2019), and have advantages such as fewer side effects and multiple targets compared to other synthetic drugs. Currently, research on polysaccharide metabolites in TCM to tonify the kidney for the treatment of osteoporosis has received widespread attention. Based on the source of polysaccharides, this review summarizes the anti-osteoporosis mechanisms of common polysaccharide metabolites of TCM for tonifying the kidney.

## 2 Literature retrieval and extraction

This review systematically searched databases including PubMed, Web of Science, and Google Scholar to comprehensively collect relevant literature from January 2006 to December 2025. The search keywords mainly consisted of “kidney-tonifying Chinese medicine,” “ginseng,” “Lycium barbarum,” “Astragalus,” “Ganoderma lucidum,” “Polygonatum,” “Morinda officinalis,” “Epimedium,” “Rehmannia glutinosa,” “Achyranthes bidentata,” “Ligustrum lucidum,” “Cynomorium songaricum,” “Eucommia,” “Dendrobium officinale,” “pharmacological effects,” “polysaccharide metabolites,” “osteoporosis,” “bone metabolism,” “osteoblasts,” and “osteoclasts.” These were combined with Medical Subject Headings (MeSH) terms, synonyms, and related expressions to optimize search strategies, thereby improving both recall and precision.

The inclusion criteria were as follows: study types primarily included randomized controlled trials (RCTs), systematic reviews, and key basic research (cell and animal experiments); the research topic must clearly address polysaccharide metabolites of herbal medicines and their pharmacological effects.

Literature screening was conducted in two stages: first, an initial screening based on titles and abstracts to exclude clearly irrelevant studies; subsequently, full texts of the remaining literature were thoroughly reviewed and evaluated in detail. Final inclusion was determined based on methodological quality, data completeness, and relevance to the review topic. Data extraction was performed independently by two researchers and cross-checked. Extracted information included basic study details, experimental design, main results, and conclusions to ensure accuracy and consistency.

## 3 Polysaccharides of TCM on anti-osteoporosis

**FIGURE 1:**
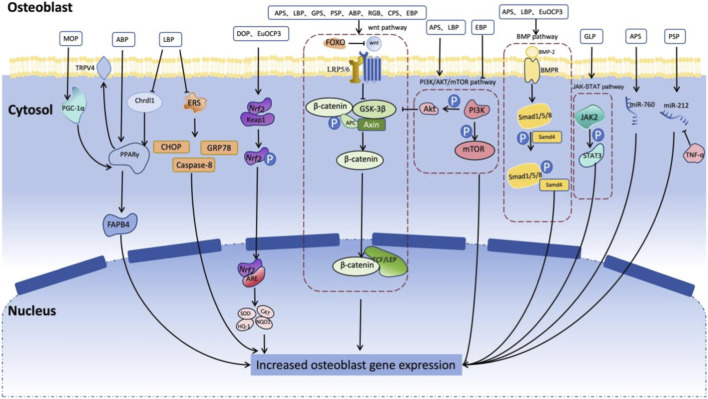
Polysaccharides from kidney-tonifying Chinese medicine affect osteoblasts. TRPV4, Transient Receptor Potential Vanilloid 4; PGC-1α, Peroxisome proliferator-activated receptor γ coactivator 1-alpha; FAPB4, Fatty acid binding protein 4; ERS, Endoplasmic Reticulum Stress; CHOP,C/EBP-homologous protein; Caspase-8, Cysteinyl aspartate specific proteinase 8; GRP78, Glucose regulated protein78kD; Nrf2, Nuclear factor-erythroid 2 related factor 2; KEAP1, Kelch-like ECH-associated protein 1; ARE, Antioxidant Response Element; SOD, Superoxide Dismutase; CAT, Catalase; NQO1,NAD (P)H: quinone oxidoreductase 1; FOXO, Forkhead box O1; wnt, Wingless/Integrated; LRP5/6, Lipoprotein Receptor-Related Protein5/6; β-catenin, β1, 88 kDa catenin (cadherin-associated protein); GSK-3β, Glycogen Synthase Kinase 3 Beta; APC, Adenomatous Polyposis Coli; PI3K, Phosphatidylinositol 3-kinase; Akt, Protein Kinase B; mTOR, Mammalian Target of Rapamycin; BMP, Bone Morphogenetic Protein; BMPR, Bone Morphogenetic Protein Receptor; JAK2, Janus Kinase 2; STAT3, Signal transducer and activator of transcription 3; TNF-α, Tumour necrosis factor-alpha.

**FIGURE 2:**
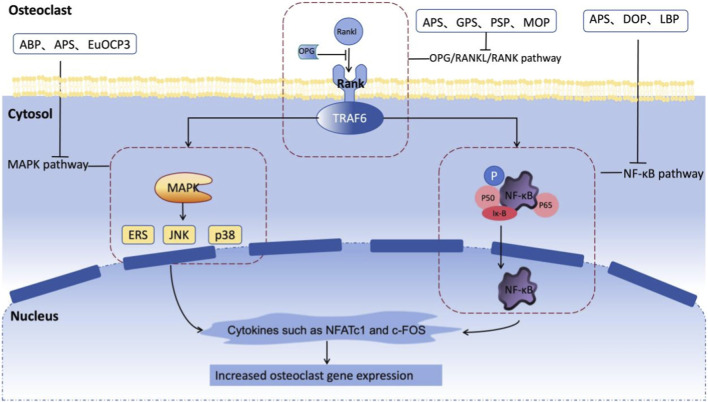
Polysaccharides from kidney-tonifying Chinese medicine affect osteoclast. Rankl, Receptor Activator of Nuclear Factor Kappa B Ligand; Rank, Receptor Activator of Nuclear Factor-κB; OPG, Osteoprotegerin; TRAF6, TNF receptor associated factor 6; MAPK, Mitogen-Activated Protein Kinase; JNK, C-Jun N-terminal kinase; p38, p38 mitogen-activated protein kinase; NF-κB, Nuclear factor kappa-B; P50, p50 protein precursor; P65, p65 protein precursor; Iκ-B, Inhibitor of NF-κB; NFATc1, Nuclear factor of activated T cells 1; c-FOS, Fos proto-oncogene, AP-1 transcription factor subunit.

This study summarized and identified 13 polysaccharide metabolites from traditional Chinese medicines with kidney-tonifying effects, and systematically analyzed their therapeutic effects on osteoporosis and the underlying mechanisms. These mechanisms were summarized from two aspects: regulating osteoblasts to affect bone formation and regulating osteoclasts to affect bone resorption, as shown in Figures 1, 2. Meanwhile, the specific mechanisms of action and pharmacological research details of these polysaccharide metabolites were compiled in Tables 1, 2.

**TABLE 1:** Mechanism of kidney in traditional Chinese medicine on bone metabolism.

Polysaccharide	Activity on bone metabolism	Specific mechanisms of action	References
APS	Promotes osteogenesis and inhibits osteogenesis	Regulation of miR-760-induces functional changes in conjunction with ANKFY1 Upregulation of the expression of BMP-2, Smad1, p-Smad1, and p-Smad5 in ovariectomized rats Inhibition of the adverse effects of oxidative stress on osteoblasts through the FoxO3a gene and enhancement of the wnt signaling pathway Upregulation of p-AKT and p-mTOR, activation of the PI3K/Akt/mTOR pathway to promote osteoblast differentiation Upregulation of the number of vitamin D receptors and regulation of the key enzymes for its activation and imbalance Upregulation of OPG and inhibition of osteoclast differentiation by suppressing the NF-kB and MAPK signaling pathways	Ren et al. (2021), Hu et al. (2023), Lowery and Rosen (2018), Zhang et al. (2021a), Che et al. (2020), Yang et al. (2016), Zhu et al. (2022), Kousteni, 2011; Ou et al. (2019), Reid et al. (2014), Cai et al. (2018a), Cai et al. (2018b), Udagawa et al. (2021), Yang et al. (2021), Huo and Sun (2016), Li et al. (2022), Sun et al. (2023), Zhou et al. (2023)
LBP	Promotes osteogenesis and inhibits osteogenesis	Inhibition of miR-200b-3p and PPARγ, upregulation of Chrdl1, and reduction of apoptosis Inhibition of ERS activation caused by the JNK pathway and reduction of osteoblast apoptosis; activation of the BMP-2/Smads signaling pathway; enhancement of the Wnt10b/β-catenin signaling pathway; inhibition of the secretion of TNF-α and IL-1β and the NF-κB signaling pathway and the expression of iNOS protein	Xu et al. (2023), Gkastaris et al. (2020), Wu et al. (2024), Jing et al. (2020), Jing and Jia (2018), Zhao et al. (2013), Liu et al. (2022), Perkins et al. (2023), Wang et al. (2017), Im and Kim (2014), Cai S. et al. (2018)
GPS	Promotes osteogenesis and inhibits osteogenesis	Downregulation of TRAP-5b, upregulation of the expression of BALP, OC and OPG, and regulation of the serum contents of P^3+^ and Ca^2+^ Upregulation of the expression of Wnt3, activation of the Wnt3/β-catenin/Runx2 signaling pathway, and promotion of osteoblast proliferation	Gao et al. (2023), Halleen et al. (2006), Li et al. (2023)
GLP	Promotes osteogenesis	In the dexamethasone-induced rat model, the upregulation of p-JAK2 and p-STAT3 protein expression was inhibited, the JAK2/STAT3 signaling pathway was regulated, and osteoblast apoptosis was inhibited Improved the bone microstructure and biomechanical properties of ovariectomized rats and treat steroid-induced osteoporosis	Xie et al. (2021), Compston (2018), Xue et al. (2023), Ge and Ge (2016)
PSP	Promotes osteogenesis and inhibits osteogenesis	Promotion of the accumulation of β-catenin in the nucleus to facilitate its binding to the transcription factors LEF/TCF and enhancement of the wnt/β-catenin signaling pathway Increases the expression of miR-212 and inhibition of osteoblast apoptosis induced by TNF-α Upregulation of the expression of genes such as Runx2, OPN, and GSK3β, enhancement of wnt, and promotion of osteogenic differentiation of osteoporosis-ASCs Upregulation of OPG, increase in RANK/OPG, and inhibition of osteoclast differentiation	Yuan and Wang (2024), Du et al. (2016), Wang and He (2020), Zhang et al. (2020), Liu C. et al. (2020), Al-Ghadban and Bunnell (2020), Lu et al. (2022), Cheng et al. (2023)
ABP	Promotes osteogenesis and inhibits osteogenesis	Downregulation of the expression of PPARγ, C/EBPα and TRPV4 mRNA and protein, inhibition of the PPARγ/TRPV4 signaling pathway to thereby inhibit the adipogenic differentiation of BMSCs; improvement of the bone microstructure of osteoporosis rats Increases levels of ALP, OC and PINP in rat serum; increase in the expression of nuclear β-catenin, Runx2 and Osteri proteins to promote the wnt/β-catenin signaling pathway Inhibition of the phosphorylation activation of JNK, ERK and p38 in the MAPK signaling pathway and the activation of downstream effector molecules such as NFATc1, proto-oncogene c-FOS protein, CTSK and integrin β3	Ha et al. (2010), Ning et al. (2022), Han et al. (2020), Yue et al. (2022), Zeng and Yang (2021), Ibáñez et al., 2022; Park (2023), Enomo et al. (2024), Sanz-Ezquerro and Cuenda (2021), Song et al. (2018)
RGP	Promotes osteogenesis	Improves the bone structure of ovariectomized rats Increases the relative expression of β-catenin, Runx2 and LRP5 proteins and affect the wnt/LRP5/β-catenin pathway	Zhang et al. (2021b), Liang et al. (2023), Xu et al. (2017), Ou et al. (2021), Kou et al. (2021)
LLPS	Inhibits osteogenesis	Regulates calcium and phosphorus metabolism Improves bone structure Downregulates the activity of TRAP enzymes and inhibit the differentiation of mononuclear cells into osteoclasts	Wang et al. (2019), Ciosek et al. (2021), Wei et al. (2022)
CPS	Promotes osteogenesis	Regulates the PI3K/Akt/GSK3β signaling pathway, increase the mRNA expression of osteogenesis-related proteins such as Runx2, β-catenin, OC, and collagen I, thereby promoting the wnt signaling pathway	Feng (2023), Karar and Maity (2011), Xu et al. (2020), Cheng et al. (2021), Hu et al. (2022)
EBP	Promotes osteogenesis	Inhibition of bax and Caspase3 and increases in the expression of Bcl2, reduction of osteoblast apoptosis, and acceleration of osteogenesis Inhibition of the PI3K/Akt/mTOR signaling pathway and activation of the wnt/β-catenin signaling pathway in osteoblasts	Guo et al. (2022), Lei et al. (2023), Zheng et al. (2020)
EuOCP3	Promotes osteogenesis and inhibits osteogenesis	Activation of the Nrf2 signaling pathway and inhibition of oxidative stress responses; inhibition of JNK phosphorylation and inhibition of osteoclast differentiation; promotion of ERK phosphorylation and induction of the ERK/BMP-2/Smad pathway to promote osteogenic differentiation	Zhou et al. (2010), He et al. (2020), Song et al. (2023), Bai et al., 2019; Song (2023)
DOP	Promotes osteogenesis and inhibits osteogenesis	Interference with the binding of KEAP1 and Nrf2, reduction of Nrf2 ubiquitination, and promotion of nuclear accumulation of Nrf2; inhibition of RANKL and its downstream genes such as NFATc1D and p38 to inhibit osteoclast differentiation	Luo et al. (2024), Wang Y. et al. (2023), Li et al. (2020)
MOP	Promotes osteogenesis and inhibits osteogenesis	Inhibition of the PGC-1α/PPARγ pathway, increase in bone mineral density, increase in trace elements in serum, and enhancement of antioxidant capacity; regulation of the cell cycle and the expression of Bcl-2 and bax, reduction of osteoblast apoptosis; regulation of the OPG/RANK/RANK signaling pathway, and reduction of osteoclast differentiation	Shen et al. (2020), Abu et al. (2023), Rong et al. (2022), Li et al. (2008), Jiao et al. (2023), Peng and Zhong (2020), Fang (2018)

**TABLE 2:** Pharmacological study on polysaccharides of traditional Chinese medicine for tonifying kidney.

Types	Species	Dose range	Model	Controls	Control group	Duration	References
APS	*Astragalus *L	100–800 mg/kg	In vivo	Positive	Nilestriol, blank	12 weeks	Ou et al. (2019), Zhou et al. (2023)
LBP	*Lycium barbarum *Lam	0–800 μg/mL	In vitro	Positive	Palmitate	1–8.5days	Jing et al. (2020), Jing and Jia (2018)
GPS	*Panax ginseng* C.A.Mey	100–200 mg/kg	In vivo	Positive	Alendronate sodium and vitamin D3	12 weeks	Li et al. (2023)
GLP	*Ganoderma lucidum* (curtis) P. Karst	2–3 mg/mL	In vitro	Positive	Blank	48 h	Xue et al. (2023)
GLP	*Ganoderma lucidum* (curtis) P. Karst	1–10 mg/kg	In vivo	Positive	Blank	12 weeks	Ge and Ge (2016)
PSP	Acanthaceae Juss	0.5–100 mg/mL	In vitro	Positive	Blank	1–7days	Du et al. (2016), Liu C. et al. (2020)
ABP	Achyranthes bidentata Blume	100–200 mg/kg	In vitro	Positive	Blank	48 h	Yue et al. (2022)
ABP	Achyranthes bidentata Blume	2–8 g/kg	In vivo	Positive	Nilestriol	12 weeks	Zeng and Yang (2021)
RGP	Rehmannia glutinosa (Gaertn.) libosch. Ex DC.	100–400 mg/kg	In vivo	Positive	Alendronate Sodium, blank	8–12weeks	Ou et al. (2021), Kou et al. (2021)
LLPS	Ligustrum lucidum W.T.Aiton	3–6 g/kg	In vivo	Positive	Estrogen	8weeks	Wei et al. (2022)
CSP	Cynomorium coccineum subsp. songaricum (Rupr.) J.Léonard	0–100 μg/m L	In vitro	Positive	Blank	7days	Hu et al. (2022)
EBP	Epimedium Tourn. Ex L	0–125 μg/mL	In vitro	Positive	Blank	24 h	Zhang J. et al. (2024), Guo et al. (2022)
EUP	*Eucommia ulmoides* Oliv	30–300 mg/kg	In vivo	Positive	Estradiol, blank	49days	Zhang J. et al. (2024), Guo et al. (2022)
DOP	Dendrobium officinale Kimura & migo	200–400 mg/kg	In vivo	Positive	Blank	8weeks	Wang Y. et al. (2023)
DOP	Dendrobium officinale Kimura & migo	25–400 μg/mL	In vitro	Positive	Blank	24 h	Li et al. (2020)
MOP	Gynochthodes officinalis (F.C.How) razafim. & B.Bremer	300–3000 mg/kg	In vivo	Positive	Blank, blank,Nilestriol	1day-12 weeks	Rong et al. (2022), Li et al. (2008), Jiao et al. (2023), Peng and Zhong (2020)

### 3.1 Astragalus polysaccharides

According to the earliest record of Astragalus in “Shennong’s Classic of Materia Medica”, it was clearly pointed out that it has the effect of “tonifying deficiency”. Additionally, Astragalus has also been recorded as tonifying the kidneys and can exert effects of “tonifying kidney yang, nourishing kidney yin, replenishing kidney essence, and transforming kidney qi”. Many active substances have been identified in Astragalus, among which the content of Astragalus polysaccharides (APS) is relatively higher than that of other metabolites, with the main components being dextran and heteropolysaccharides (Ma et al., 2022).

#### 3.1.1 APS for osteoporosis

The upstream gene miR-760 of Rabankyrin-5 (ANKFY1), which contains ankyrin repeats and FYVE domains, was recently found to be upregulated in osteoporosis tissues and downregulated during osteogenic differentiation of human BMSCs. Silencing of the miR-760 target gene can increase bone mineral density in osteoporosis rats, thus alleviating the progression of osteoporosis (Ren et al., 2021). Hu et al. (2023) found that APS promoted osteogenic differentiation and proliferation of human BMSCs. Upon assessing the levels of miR-760 and ANKFY1 in groups treated with APS, it was hypothesized that an interaction between ANKFY1 and its upstream regulator miR-760 may exist. Specifically, ANKFY1 might be involved in modulating the functional changes triggered by miR-760 in APS-treated BMSCs, thus facilitating osteogenic differentiation and proliferation of BMSCs and conferring an anti-osteoporotic effect.

Bone morphogenetic protein (BMP) depends on two signaling pathways: the Smad and the p38 mitogen-activated protein kinase (p38 MAPK) pathways. Its activation enhances the proliferation of mouse skeletal stem cells, and BMP has a sufficient application potential in the field of bone and cartilage regeneration (Lowery and Rosen, 2018). Zhang et al. (2021a) found that APS treatment reversed the decreased expression of BMP-2, Smad1, phosphorylated (p)-Smad1, and p-Smad5 in ovariectomized rats. Therefore, APS is believed to exert its effects by stimulating activation of the BMP-2/Smad signaling pathway. After activation of the Smad signaling pathway, it stimulates osteogenic differentiation of BMSCs and reduces the number of osteoclasts in rats, thus exerting an anti-osteoporosis effect.

Iron metabolism disorders not only promote osteoclast differentiation and osteoblast apoptosis, but also inhibit osteoblast proliferation and differentiation, ultimately disrupting the bone metabolism homeostasis. The density and strength of the bones also change, increasing the probability of the occurrence of osteoporosis (Che et al., 2020). Yang et al. (2016) found that in the iron overload model induced by ammonium ferric citrate (FCA), APS alleviated the decrease in BMSC viability, proliferation inhibition, apoptosis, and cell senescence.

By detecting the level of reactive oxygen species (ROS), an oxidative stress product, ROS-mediated mitochondrial dysfunction is involved in FAC-induced BMSC apoptosis, and APS treatment improved this apoptosis. Oxidative stress is an independent factor that mediates bone homeostasis dysfunction, increases osteoclastogenesis, reduces the differentiation of bone precursor cells into osteoblasts, and leads to decreased osteoblast activity and increased apoptosis (Zhu et al., 2022). Therefore, inhibition of the oxidative stress response is a way to alleviate osteoporosis.

The activation of members of the FoxO family is an important factor that induces oxidative stress and can affect the function of the canonical Wnt/β-catenin pathway, inhibiting the differentiation of BMSCs into osteoblasts (Kousteni, 2011). Ou et al. (2019) found that APS can inhibit FoxO3a gene expression, increase the gene expression of Wnt2, β-catenin and LDL receptor-related protein 5 (LRP5) mRNA, inhibit the FoxO3a/Wnt2/β-catenin signaling pathway, enhance the Wnt/LRP5/β-catenin signaling pathway, inhibit the appearance of oxidative stress, and promote osteogenic differentiation of BMSCs.

Vitamin D is crucial for maintaining the balance of calcium and phosphorus metabolism. The intake of an appropriate amount of vitamin D enhances Ca^2+^ absorption, which is beneficial for protecting bone health. Several studies have reported a correlation between vitamin D levels and osteoporosis. The concentration of vitamin D and the number of vitamin D receptors have important influences on osteoporosis (Reid et al., 2014). Cai et al. (2018a) found that APS upregulates the expression of vitamin D receptor mRNA and related proteins in osteoblasts. Under stimulation of a certain concentration of APS, the number of vitamin D receptors increased, which was conducive to an improvement in osteoblast activity. Further studies (Cai et al., 2018b) found that APS also upregulates and downregulates two key enzymes, cytochrome P450 27B (CYP27B) and cytochrome P450 24A (CYP24A), which play key roles in the activation and metabolism of vitamin D, respectively.

Osteoclasts regulate bone resorption. Abnormally high osteoclast activity causes bone resorption to exceed bone formation for an extended period, leading to the onset of osteoporosis. Osteoclast formation and differentiation are inextricably linked the key cytokine receptor activator of NF-κB ligand (RANKL) (Udagawa et al., 2021). Yang et al. (2021) found that APS affects osteoclast formation by inhibiting the receptor activator of NF-κB ligand (RANKL)-stimulated mitogen-activated protein kinase (MAPK) signaling pathways, including extracellular signal-regulated kinase (ERK), c-Jun N-terminal kinase (JNK), and p38. Conversely, APS inhibits the production of RANKL-stimulated intracellular reactive oxygen species (ROS), and ROS is related to the effect of activating the downstream NF-kB signaling pathway and further activates osteoclast-related genes. In summary, APS showed inhibitory effects on MAPK and ROS signaling pathways, ROS production, and downstream factors responsible for osteoclastogenesis. Huo and Sun (2016) reported that APS regulates RANKL/OPG by inhibiting the expression of RANKL and increasing osteoprotegerin (OPG), enhancing the binding of OPG to RANKL, and reducing the binding of RANK and RANKL, thus affecting the expression of downstream genes to inhibit osteoclast differentiation.

The PI3K/Akt/mTOR signaling pathway, which includes Phosphoinositide 3-kinase (PI3K), the downstream mediator protein kinase B (Akt), and the mammalian target of rapamycin (mTOR), plays a crucial role in regulating the proliferation and differentiation of osteoblasts and osteoclasts (Li et al., 2022). Some studies (Sun et al., 2023) have indicated that APS activates the PI3K/Akt/mTOR signaling pathway. When PI3K/Akt/mTOR was inhibited, APS treatment did not significantly affect PI3K, AKT, or mTOR; however, the expression of p-AKT and p-mTOR increased, thus improving osteoblast proliferation and differentiation ability and inhibiting osteoclast differentiation.

Zhou et al. (2023) found that compared to APS alone treatment, APS involved in treatment after fermentation by *Lactobacillus* acidophilus (LA), the osteoclast differentiation marker acid phosphatase 5 (ACP5) decreased more significantly, the osteoblast differentiation marker osteocalcin (OCN) increased more significantly, and also significantly reduced the levels of pro-inflammatory cytokines tumor necrosis factor α (TNF-α) and interleukin (IL)-6. In the osteoporosis rat model, combined LA and APS treatment significantly restored calcium absorption in osteoporosis rats, which was conducive to improving bone density and microstructure. This indicates that after LA fermentation, APS is more effective in inhibiting osteoclast differentiation and promoting osteoblast differentiation and is helpful for the recovery of osteoporosis. LA is typically found in the human intestine. The therapeutic role of the combination of APS and LA fermentation in the human body and the mechanisms underlying its synergistic action require further exploration in future studies.

#### 3.1.2 Shortcomings of APS application

Currently, APS is primarily applied in anti-tumor therapy, and research in this area is well-established (He et al., 2024). Its clinical use in veterinary medicine has also been reported (Xue, 2023). However, there remains scarce reporting on its human clinical applications in the field of bone metabolism.

### 3.2 Lycium barbarum polysaccharide

Lycium barbarum is a traditional Chinese medicinal plant. There are similar records indicating its properties such as “robust sexual function”, “nourishing the liver and kidneys,” “strengthening the bones and tendons, resisting cold and heat, and remaining youthful” in various books. It is generally believed that the main effect of Lycium barbarum is the nourishment of the kidney yin. Lycium barbarum polysaccharide (LBP) is composed of four key metabolites: galactose, arabinose, glucose, and glucosamine (Xu et al., 2023).

#### 3.2.1 LBP for osteoporosis

In recent years, obesity has been shown to be an important factor in the generation of osteoporosis (Gkastaris et al., 2020). Peroxisome proliferator activated receptors γ (PPARγ) can inhibit the Wnt/β-catenin signaling pathway and OPG secretion, affecting osteoblast formation and differentiation (Wu et al., 2024). At the same time, it induces the expression of the CCAAT-enhanced binding protein (CCAAT enhanced binding protein, CEBP), especially enhancing the expression of the CEBPα gene and promoting the generation of adipocytes. For obese patients, this effect of PPARγ may further aggravate their weight and bone problems. The BMP antagonist Chordin-like-1 (CHRDL1), a target of the miR-200a family, participates in osteoclast differentiation and plays a crucial role in osteoblast apoptosis. Jing et al. (2020) found that in an obese environment simulated by palmitic acid (PA) induction *in vivo*, PA can partially upregulate the expression of miR-200b-3p and PPARγ, thereby specifically inhibiting Chrdl1 expression and promoting the apoptosis of osteoblasts. After LBP treatment, miR-200b-3p expression was directly downregulated, Chrdl1 expression was directly upregulated and PPAR expression was indirectly inhibited, thus reducing osteoblast apoptosis and promoting osteoblast proliferation and differentiation. However, the mechanism through which LBP protects osteoblasts from palmitate-induced damage remains unclear. Chemical stimulation can cause changes in the function of the endoplasmic reticulum, known as the endoplasmic reticulum stress response (ERS). Persistent and excessive ERS activates the homologous protein of the CCAAT/enhancer binding protein (CHOP), cysteinyl aspartate-specific proteinase-12 (caspase-12), and the JNK pathway, leading to cell apoptosis. Jing and Jia (2018) proof, PA activation of the CHOP, caspase-12, and JNK pathways significantly increased ERS-induced cell apoptosis. After LBP treatment, the expression of the most sensitive signals, glucose-regulated protein 78 (glucose regulated protein 78, GRP78), CHOP and caspase-12 in ERS and the phosphorylation level of JNK were effectively reduced, indicating that LBP can also reduce osteoblast apoptosis by inhibiting ERS activation caused by the JNK pathway. Zhao et al. (2013) found that LBP had a therapeutic effect in promoting fracture healing. It has been speculated to promote the expression of BMP-2 and that the effect of LBP in promoting the expression of BMP-2 has a certain dose-effect relationship and a duration-effect relationship of administration. Increasing the expression of BMP-2 may stimulate the BMP-2/Smad signaling pathway, thus promoting the proliferation of BMSCs and their transformation into osteoblasts.

The classical Wnt signaling pathway activates gene transcriptomes and facilitates the transfer of extracellular signals to the nucleus, thereby exerting many important biological functions. In the presence of Wnt, when the cytoplasmic phosphoprotein Disheveled (Dsh/Dvl) binds to the N-terminal extracellular cysteine-rich domain of the Frizzled (Fz) receptor family, β-catenin is degraded after the Wnt/β-catenin signaling pathway is activated (Liu et al., 2022). The Wnt10b signaling pathway promotes osteoblast differentiation and reduces adipocyte differentiation by promoting β-catenin and inhibiting PPARγ, thereby promoting bone formation (Perkins et al., 2023). Wang et al. (2017) found that LBP can increase the expression of β-catenin and Wnt10b, suggesting the enhancement of the Wnt10b/β-catenin signaling pathway, proving that LBP can increase the expression of Wnt10b, thus inhibiting adipocyte formation and increasing osteoblast formation, exerting an anti-osteoporosis effect.

Osteoporosis increases the risk of osteoarthritis (OA), and osteoarthritis can increase the risk of osteoporosis. Most patients with osteoarthritis have osteoporosis, which accelerates the progression of OP (Im and Kim, 2014). Cai S. et al. (2018) found that 400 μg/mL of LBP can inhibit the secretion of TNF-α and IL-1β in cells and downregulate the expression of iNOS and NF-κB p65 protein, and it has a certain inhibitory effect on the inflammatory response to OA.

#### 3.2.2 Shortcomings of LBP application

LBP has shown certain applications in anti-tumor therapy, ophthalmological diseases, and oral diseases, its use in the field of bone diseases remains confined to basic research (Chen et al., 2018).

### 3.3 Ginseng polysaccharide

Ginseng, often hailed as the “King of medicinal plant,” is recorded to have the functions of “improving physical fitness and prolonging life”, “replenishing vital energy greatly” and “treating deficiency syndromes.” Ginseng polysaccharide (GPS) is the main active component and contains five monosaccharides: rhamnose, galacturonic acid, glucose, galactose, and arabinose. The glucose content exceeds half of the total glucose content (Gao et al., 2023).

#### 3.3.1 GPS for osteoporosis

Tartrate-resistant acid phosphatase (TRAP), a key enzyme in bone metabolism, exists in two forms: TRAP-5a and TRAP-5b. Primarily localized in osteoclasts as TRAP-5b, it is responsible for the majority of the TRAP activity detected in serum. Consequently, the levels of TRAP, especially TRAP-5b, can be used as an index of bone resorption and can provide clues regarding bone metabolism in various physiological and pathological states (Halleen et al., 2006). In the study by Li et al. (2023), after establishing an ovariectomized osteoporosis rat model, it was found that the bone trabeculae of the rats in each GPS dose group were closer to normal compared to the sham operation group and the model group; compared to the model group, the bone mineral density of the rats in the low and high dose GPS groups also increased accordingly, and the bone strength, maximum stress, and maximum load of rats increased significantly. The results of the detection of serum markers showed that the serum content of TRAP-5b content in rats in each GPS dose group decreased, whereas the content of bone-specific alkaline phosphatase (BALP), osteocalcin (OC), OPG, P^3+^ and Ca^2+^ increased. Among these, BALP and OC are important indicators of osteoblast formation. This indicates that GPS can regulate the activities of osteoclasts and osteoblasts and increase the mineral salt content of the bone. Furthermore, the research results also found that the expression of Wnt3, β-catenin and Runt-related transcription factor 2 (Runx2) in the bone tissue of rats in each GPS dose group increased significantly, indicating that it can stimulate osteogenesis by activating the Wnt3/β-catenin/Runx2 signaling pathway to counteract osteoporosis.

#### 3.3.2 Shortcomings of GPS application

GPS can be applied in the treatment of tumors, diabetes mellitus, viral hepatitis, and sepsis, among other diseases. However, in the field of bone diseases, its application remains confined to basic research and has not yet entered clinical use (Yang et al., 2023).

### 3.4 Ganoderma polysaccharide

Ganoderma lucidum, revered as a traditional medicinal material, often features as an “immortal medicine” in Taoism prescriptions. It is documented to possess functions such as ‘prolonging life and attaining immortality’, ‘benefitting the waterways, tonifying kidney qi’, ‘benefitting essence qi and strengthening muscles and bones’. There are many types of Ganoderma polysaccharide (GLP), which are extremely rich, with more than 200 known varieties. Monosaccharide metabolites are also abundant, mainly glucose and galactose, with small amounts of arabinose and xylose (Xie et al., 2021).

#### 3.4.1 GLP for osteoporosis

Excessive use of glucocorticoids is the most common cause of secondary osteoporosis (Compston, 2018), which may be associated with their effect on osteoclast activity and osteoblast apoptosis. The Janus kinase (JAK)/signal transducer and activator of transcription (STAT) signaling pathway consists of three key metabolites: one is the receptor that can receive upstream signals, the second is the intermediate molecule that transmits signals, namely, the tyrosine kinase of the JAK family and the transcription factor STAT family, which finally responds to the signal and exerts its effect. These three metabolites work together to form a complete JAK/STAT signal transduction pathway and jointly participate in the regulation of physiological processes such as cell growth, differentiation, and survival. In the study by Xue et al. (2023), after treating osteoblasts with 1 μmol/L glucocorticoid analog dexamethasone to construct an osteoporosis cell model, treatment with treatment with dexamethasone increased the expression and activity of the BCL2-associated X protein (Bax) and Caspase-3, and decreased the expression of the anti-apoptosis-related protein B-cell lymphoma-2 (Bcl-2), promoting apoptosis of osteoblasts MC3T3-E1. After GLP treatment, these effects were reversed to various degrees, inhibiting upregulation of Bax and caspase-3 and downregulation of Bcl-2. Furthermore, GLP treatment inhibited upregulation of the expression of the dexamethasone-induced p-JAK2 and p-STAT3 proteins, indicating that GLP can regulate key pathways related to bone formation, such as the JAK2/STAT3 signaling pathway, and inhibit apoptosis of MC3T3-E1 osteoblasts. Ge and Ge (2016) found that the Taishan Ganoderma lucidum polysaccharide extract (with a GLP content of 20%) can improve osteoporosis in ovariectomized female Wistar rats. The bone diameter and volume of each dose group of the Taishan ganoderma lucidum polysaccharide extract increased to varying degrees, and bone length, wet weight and dry weight, significantly compared to the ovariectomized group, indicating that the Taishan ganoderma lucidum polysaccharide extract has a promoting effect on bone formation in hormone-induced osteoporosis rats, thus treating hormone-induced osteoporosis.

#### 3.4.2 Shortcomings of GLP application

GLP hold promising clinical application prospects, with extensive basic research conducted in areas such as antitumor activity, antioxidant effects, immunomodulation, and bone metabolism. However, none of these applications have yet progressed to clinical use (Zhong et al., 2024).

### 3.5 Polygonatum polysaccharide

Polygonatum sibiricum, celebrated as both a medicinal and edible medicinal plant, is recognized for its abilities to “nourish the kidneys and filling the essence” and “strengthening tendons and bones”. Polygonatum polysaccharide (PSP) are an active component of Polygonatum sibiricum, consisting mainly comprising mannose, glucose, galactose, fructose, galacturonic acid, arabinose, and glucuronic acid (Yuan and Wang, 2024).

#### 3.5.1 PSP for osteoporosis

In the classic Wnt/β-catenin pathway, stable β-catenin is transported to the nucleus by Ras-related C3 botulinum toxin substrate 1 (Rac1) and other factors. Within the nucleus, it binds to the transcription factor of the free cell T cell factor (TCF)/lymphoid enhancer factor (LEF), replaces the co-repressor, recruits more co-activators and further activates the expression of downstream target genes of the Wnt/β-catenin pathway. Du et al. (2016) treated BMSCs with PSP for 24, 48, and 72 h, and total cell RNA was extracted to detect the expression of β-catenin mRNA, it was observed that PSP could enhance the accumulation of β-catenin in the nucleus and its binding to the transcription factor LEF/TCF, thus activating the expression of downstream target genes of the Wnt/β-catenin signaling pathway, indicating that PSP could enhance the transcriptional activity of β-catenin/TCF. Not only that, when detecting downstream transcription factors Smad1/5/8 of BMP, it was noted that PSP appears to exert no significant influence on the BMP signaling pathway.

TNF-α can promote and inhibit osteoblast differentiation, which may be related to concentration, duration of action, and target cells (Wang and He, 2020). miR-212 is to promote osteoblast proliferation and reduce apoptosis. Recent research findings suggest that it is possible to effectively treat osteoporosis by activating the OPG/RNKL pathway and upregulating RUNX2 (Zhang et al., 2020). Liu C. et al. (2020) found that TNF-induced osteoblasts could lead to downregulation of miR-212 levels. However, the addition of PSP reversed the trend changing miR-212 levels, thus increasing the expression of Cyclin D1 and BcL-2 and reducing the expression of the Bax and P21 proteins. To further confirm the mechanism by which PSP exerts its effects, after experimentally inhibiting miR-212 expression, we found that the addition of PSP addition did not improve osteoblast apoptosis, and there were no significant changes in the content of various proteins in osteoblasts. Therefore, it can be considered that the effect of improving PSP on apoptosis of osteoblasts induced by TNF-α is exerted by increasing miR-212 expression. However, the mechanism by which miR-212 regulates the expression of these proteins requires further investigation.

Adipose-derived stem cells (ASCs), akin to BMSCs, are capable of differentiating into osteoblasts, chondrocytes, and adipocytes. Owing to their strong regenerative potential, they have recently become candidates for regenerative medicine and the treatment of inflammatory diseases (Al-Ghadban and Bunnell, 2020). Lu et al. (2022) confirmed that after the intervention of PSP in ASC, Runx2 and osteopontin protein and mRNA expression of Runx2 and osteopontin (OPN) increased significantly, markedly improving the osteogenic differentiation ability of ASC. By examining the protein expression of β-catenin and glycogen synthase kinase 3β (GSK3β) and the mRNA expression of β-catenin, higher expression of β-catenin and reduced the decomposition effect of GSK3β on β-catenin were observed, thereby enhancing the Wnt/β-catenin signaling pathway and promoting osteogenic differentiation of osteoporosis-ASCs.

Cheng et al. (2023) demonstrated that the serum Ca^2+^ and OC content in groups treated with varying doses of PSP was significantly lower compared to the diabetic osteoporosis model group. Serum ALP, TRAP, and P^3+^ were significantly lower in the high-dose PSP group than those in the model group for diabetic osteoporosis model group. In bone tissue protein detection, the level of RANKL protein in each PSP group decreased, whereas that of OPG protein increased, affecting RANKL/OPG/RAK to inhibit osteoclast formation.

#### 3.5.2 Shortcomings of PSP application

PSP have been studied in various fields, including antioxidant activity, immune regulation, antitumor effects, anti-osteoporosis, hypoglycemia, lipid-lowering, and anti-atherosclerosis. However, most research in the area of bone metabolism remains at the experimental stage and has not yet entered clinical application (Guo et al., 2025).

### 3.6 Achyranthes bidentata polysaccharide

Achyranthes bidentata, commonly found on the edges of forests and among grasses in hillsides, is recognized for its traditional medicinal properties. It is recorded to have the functions of “filling bone marrow”, “preventing white hair” and “benefitting essence”, and it is a commonly used Chinese herbal medicine for treating lumbar and leg pain and arthritis. Achyranthes bidentata polysaccharides (ABP) are bioactive sugars that consist mainly of three monosaccharide metabolites: glucose, fructose, and mannose. Notably, the fructose content is approximately eight times higher than that of glucose (Ha et al., 2010).

#### 3.6.1 ABP for osteoporosis

Abnormal osteogenic and adipogenic differentiation of BMSCs can lead to diseases such as osteoporosis (Ning et al., 2022). The molecular mechanism underlying adipogenic differentiation of BMSCs is intricate. PPARγ regulates adipocyte differentiation and development, and its downstream CCAAT enhancer-binding protein-α (C/EBPα) is an early transcription factor that induces adipocyte formation. The transient receptor potential vanilloid 4 (TRPV4) channel is involved in fat metabolism. TRPV4 mutations can promote osteogenic differentiation of stem cells and their activation may be related to adipogenic differentiation (Han et al., 2020). Yue et al. (2022) reported that following treatment with the PPARγ agonist rosiglitazone, the expression of PPARγ, C/EBPα, and TRPV4 mRNA and protein in BMSCs increased significantly, indicating that PPARγ was activated, thus upregulating the downstream expression of C/EBPα and TRPV4. The expression of PPARγ, C/EBPα, and TRPV4 mRNA and protein in cells treated with ABPS were significantly and the PPARγ/TRPV4 pathway was significantly inhibited. After the combined treatment of the two, the promoting effect of rosiglitazone on the transcription of related genes and protein expression was reversed, confirming the inhibitory effect of ABPS on the PPARγ/TRPV4 pathway, that is, ABPS can inhibit adipogenic differentiation of BMSCs and can alleviate osteoporosis caused by abnormal differentiation of BMSCs to some extent.

Zeng and Yang (2021) reported that after 12 weeks of treatment in each ABP dose group and the positive control group, the bone mineral density significantly increased and bone metabolism was markedly improved. Alkaline phosphatase (ALP), OC, and type I procollagen N-terminal propeptide (PINP) in the serum of rats in the ABP group were higher than those of the model group, indicating that ABP can promote bone formation. At the same time, it was found that after treatment with ABP, the protein expression of β-catenin, nuclear β-catenin, Runx2 and Osteri increased, whereas that of p-β-catenin decreased, suggesting that the improvement of bone metabolism level by ABP may be associated with the activation of the Wnt/β-catenin signaling pathway.

Nuclear factor-activated T cell 1 (NFATc1) is regulated by the upstream RANKL signaling pathway. When RANKL binds to RANK, it triggers the dephosphorylation of NFATc1, thus regulating thus the expression of osteoclast-related genes, such as cathepsin K (CTSK) and Integrin β3 (Ibáñez et al., 2022). MAPK consist of three main subfamilies: ERK, JNK, and p38/MAPK. The ERK signaling pathway is involved in cell proliferation and differentiation (Park, 2023). The JNK family is an important signal transduction molecule for cells in response to various stress stimuli, such as radiation and changes in osmotic pressure, which regulate cells’ responses to these stress responses (Enomo et al., 2024). The p38 pathway is involved primarily in the regulation of the inflammatory response and apoptosis (Sanz-Ezquerro and Cuenda, 2021). The main MAPK pathways have clear divisions of work, jointly maintain cellular physiological functions, and respond synchronously to various internal and external environmental signals. Song et al. (2018) found that in bone marrow monocytes and bone marrow macrophages, ABP at a concentration of 10 μmol/L could inhibit the phosphorylation and activation of the three main MAPK proteins JNK, ERK, and p38 induced by the RANK signaling molecule RANKL, and also inhibit the activation of downstream effector molecules such as NFATc1, proto-oncogene c-FOS protein, CTSK and Integrin β3. This indicates that ABP may inhibit the formation and differentiation of osteoclasts by inhibiting the MAPK and NFATc1 signaling pathways.

#### 3.6.2 Shortcomings of ABP application

ABP exhibits broad pharmacological effects, including anti-tumor, antioxidant, anti-osteoporosis, and anti-inflammatory activities. However, these effects are currently confined to the research stage (Chai et al., 2024).

### 3.7 Rehmannia glutinosa polysaccharide

There are two forms of Rehmannia: raw and prepared. Raw Rehmannia is believed to “nourish kidney yin” while prepared Rehmannia is thought to “tonify kidney essence”. Rehmannia glutinosa polysaccharides (RGP) consist of various monosaccharides, such as rhamnose, glucose, galactose, mannose, xylose, and arabinose, and the types and proportions of monosaccharides in different Rehmannia polysaccharides vary greatly (Zhang et al., 2021b). RGP have demonstrated strong antioxidant capabilities in animal studies, exhibiting anti-aging and anti-tumor effects (Liang et al., 2023; Xu et al., 2017).

#### 3.7.1 RGP for osteoporosis

Ou et al. (2021) assessed the histomorphometric parameters of static and dynamic bone. RGP significantly increased the percentage of area of the trabecular bones, the number of trabecular bones, and the width of the trabecular bones and significantly reduced the trabecular separation in ovariectomized rats. Dynamic bone histomorphometric parameters measurement showed that RGP significantly increased bone tissue volume, bone formation rate, and bone mineralization deposition rate and significantly reduced the number of osteoclasts per millimeter in ovariectomized rats, demonstrating that RGP can promote bone formation, inhibit bone resorption, improve bone structure and treat osteoporosis after ovariectomy. Kou et al. (2021) reported that the relative expression of β-catenin, Runx2, and LRP5 in the bone tissue of rats in each dose group of rehmannia polysaccharide increased significantly, and the effect of the high-dose group was better than that of the low-dose group, suggesting that RGP may affect the Wnt/LRP5/β-catenin pathway and activate Runx2 expression of Runx2 to regulate osteoblast proliferation and differentiation and improve bone metabolism in diabetic rats.

#### 3.7.2 Shortcomings of RGP application

Rehmannia glutinosa polysaccharides show promising application prospects in areas such as immunomodulation, anti-tumor activity, antioxidant effects, and improvement of bone metabolism. However, most of these studies remain at the experimental stage and have not yet been widely applied in clinical practice (Jia et al., 2023; Bian et al., 2023).

### 3.8 Ligustrum lucidum polysaccharide

Ligustrum lucidum is renowned for its abilities to “strengthening the waist and knees,” “changing white hair,” and “benefiting the kidneys.” The main metabolites are Ligustrum lucidum polysaccharide (LLPS), L. lucidum phenol, L. lucidum glycoside, and L. lucidum acid. LLPS has a relatively high content in the water extract of ligustrum lucidum, and is composed of four monosaccharides, namely, glucose, rhamnose, arabinose, and mannose, in order of content (Wang et al., 2019). Recently, LLPS was found to play a role in alleviating osteoporosis.

#### 3.8.1 LLPS for osteoporosis

The balance of bone metabolism is intricately related to the metabolism of calcium and phosphorus in the body. Ca^2+^ levels of Ca^2+^ in the serum of patients with osteoporosis usually increase significantly, while P^3+^ levels in the serum often decrease (Ciosek et al., 2021). Therefore, regulation of calcium and phosphorus metabolism is of great significance for maintaining the balance of bone metabolism and contributing to the treatment of osteoporosis. Wei et al. (2022), using an osteoporosis mouse model, established that different doses of LLPS significantly reduced the level of Ca^2+^ in the plasma of mice, and 6 g/kg LLPS significantly reduced the content of P^3+^ in the plasma of mice. The number of trabecular bones and the fraction of bone volume in the 3 g/kg LLPS group increased significantly and trabecular separation decreased significantly, which markedly improved bone loss and damage to bone microstructure caused by castration. TRAP enzyme activity in the culture supernatants of the 1 × 10^−3^, 1 × 10^−4^, and 1 × 10^−5^ mg/mL LLPS groups decreased significantly, indicating that LLPS inhibited the activity of osteoclasts, which may be related to the inhibition of osteoclast differentiation. of bone marrow-derived mononuclear cells into osteoclasts. Therefore, LLPS may play an antiosteoporotic role by regulating calcium and phosphorus metabolism and inhibiting osteoclast differentiation. However, the mechanism by which LLPS affects the metabolism of calcium and phosphorus is unclear, and whether there are additional side effects requires further exploration.

#### 3.8.2 Shortcomings of LLPS application

In the field of bone metabolism, Ligustrum lucidum polysaccharides can inhibit osteoclast differentiation, regulate calcium and phosphorus metabolism, and alleviate osteoporosis symptoms in ovariectomized mice. However, current research on Ligustrum lucidum polysaccharides in bone metabolism remains largely confined to animal studies and has not yet been widely applied in clinical practice (Liu S. et al., 2020).

### 3.9 Cynomorium songaricum polysaccharide

Cynomorium songaricum, native to desert regions, has earned the moniker “elixir of immortality”. It is reputed for its abilities to “benefitting essence and promoting yang’ and “filling essence and consolidating the true’. Cynomorium Songaricum Polysaccharides (CSP) is a polysaccharide derived from *Candida* songaricum. Its main monosaccharide metabolites include glucuronic acid, glucose, and arabinose, of which arabinose is present in a relatively high proportion (Feng, 2023).

#### 3.9.1 CSP for osteoporosis

The PI3K/Akt pathwayplays a crucial role in the regulation of various biological processes and cellular mechanisms and regulates the proliferation and apoptosis of different cells (Karar and Maity, 2011; Xu et al., 2020). When PI3K binds to the growth factor receptor, it changes and activates the protein structure of activated Akt. Activated Akt phosphorylates GSK3β, reduces its degradation effect on β-catenin, regulates the Wnt/β-catenin pathway (Cheng et al., 2021), and promotes osteoblast formation. Hu et al. (2022) found that CSP promotes the expression of mRNA of osteogenesis-related proteins such as Runx2, β-catenin, and OC, facilitates the presence of β-catenin in the nucleus and promotes osteogenic differentiation of cells. After conducting the Western blot experiment, it is speculated that the effect of CSP on bone metabolism may play a role by affecting the PI3K/Akt/GSK3β/β-catenin pathway. After adding the PI3K inhibitor, the changes in mRNA levels of these proteins were reversed, which also verified that the mechanism by which CSP affects osteoblast proliferation and differentiation may be related to the downstream pathway of PI3K.

#### 3.9.2 Shortcomings of CSP application

CSP shows promising application potential in the field of bone metabolism. It can enhance the antioxidant capacity of osteoblasts and protect them against oxidative stress damage induced by homocysteine by activating the Keap1/Nrf2 signaling pathway, demonstrating anti-osteoporosis potential. However, current research on CSP in bone metabolism remains largely at the basic research stage, and extensive clinical studies have not yet been conducted (Zhang J. et al., 2024).

### 3.10 Epimedium polysaccharide

Epimedium is a commonly used TCM in clinical practice and is recorded to have the functions of “warming and tonifying the kidney yang” and “invigorating the yang and generating essence”. Epimedium polysaccharide (EBP) is one of the main metabolites of the epimedium and is composed of three metabolites: EBP-1, EBP-2, and EBP-3. It is mainly composed of monosaccharides such as mannose, glucose, galactose, and arabinose, among which the galactose content is the highest (Guo et al., 2022).

#### 3.10.1 EBP for osteoporosis

The interaction between Bax and Bcl2 is modulated by EBP. Bcl-2 is an antiapoptotic protein, whereas Bax is a pro-apoptotic protein. Bcl-2 blocks cell death by preventing Bax activation and homo-oligomerization. However, Bcl-2 and Bax may regulate cell proliferation, differentiation and death (Ciosek et al., 2021). The apoptotic protein caspase-3 mainly functions by cleaving nuclear substrates. Lei et al. (2023) found that EBPC1 promotes osteoblast proliferation induced by high glucose levels and affects the differentiation and mineralization of osteoblasts induced by high glucose levels, which can depend on the influence of EBPC1 on Bax/Bcl-2 expression. This study found that EBPC1 inhibited Bax and caspase3 and increased the expression of Bcl2. EBPC1, as a Bax inhibitor, increases ALP and Runx2 mRNA levels of ALP and Runx2. EBPC1 at a concentration of 100 μg/mL, like the Bax inhibitor, significantly increases the protein level of Bcl2. Therefore, the treatment with EBP and EBPC1 can improve apoptosis by regulating Bax/Bcl-2 and accelerating osteogenesis in osteoblasts. Zheng et al. (2020) and other studies also found that EPB, in the process of regulating the Bax and Bcl-2 proteins to exert antiapoptosis on osteoblasts, plays a role in inhibiting the PI3K/Akt/mTOR signaling pathway, and also has the role of activating the Wnt/β-catenin signaling pathway of osteoblasts, promoting osteoblast proliferation and differentiation.

#### 3.10.2 Shortcomings of EBP application

In terms of clinical applications, Epimedium polysaccharides exhibit antioxidant, anti-osteoporotic, immunomodulatory, and antitumor effects. Particularly in the field of anti-osteoporosis, they can promote osteoblast differentiation and inhibit osteoclast activity by activating the Wnt/β-catenin signaling pathway. However, current research on Epimedium polysaccharides remains largely at the experimental stage and has not yet been widely applied in clinical practice (Wu et al., 2025).

### 3.11 Eucommia ulmoides polysaccharide

Eucommia in TCM often refers to the bark of the Eucommia ulmoides plant, which tonifies the liver and kidneys” and” strengthens muscles and bones”. Through the extraction and hydrolysis of free sugar and polysaccharide metabolites and analysis by mass spectrometry, it can be concluded that there are monosaccharide metabolites, such as xylose, ribose, arabinose, and glucose (Zhou et al., 2010).

#### 3.11.1 Eucommia ulmoides polysaccharide for osteoporosis

Nuclear factor erythroid 2-related factor 2 (Nrf2) is widely present in most cells, and its downstream signaling pathways are important mechanisms against oxidative stress (He et al., 2020). Song et al. (2023) found that a functional Eucommia ulmoides polysaccharide (EUP), namely, EuOCP3, is an acidic polysaccharide purified from Eucommia ulmoides Oliver cortex, extracted from the Eucommia ulmoides cortex, can activate the Nrf2 signaling pathway and upregulate the expression of downstream genes such as NQO1, SOD, and CAT, thereby inhibiting oxidative stress and enhancing osteogenic function. Another study found that EuOCP3 inhibited the phosphorylation of JNK in the MAPK pathway, thus inhibiting oxidative stress related to bone resorption. However, the phosphorylation of ERK, another MAPK signaling pathway, increased, which may be related to the role of ERK in activating the nuclear accumulation and transcriptional activity of Nrf2 (Bai et al., 2019). A similar study by Song (2023) found that increased phosphorylation of ERK1/2 induced by EuOCP3 was accompanied by increased phosphorylation of Smad1/5/8, which became increasingly significant with an increase in osteogenic induction time. When the ERK signaling pathway was inhibited, the levels of phosphorylation of ERK1/2 and Smad1/5/8 and the expression of osteogenic differentiation-related proteins, including BMP-2 and Runx2, were inhibited, suggesting that EuOCP3 can promote osteogenic differentiation by inducing the ERK/BMP-2/Smad pathway.

#### 3.11.2 Shortcomings of EUP application

In terms of clinical applications, Eucommia ulmoides polysaccharides exhibit various pharmacological activities such as immunomodulation, bone protection, hypoglycemic and lipid-regulating effects, anti-inflammatory and analgesic properties, antioxidant activity, anti-fatigue effects, and anti-aging potential. However, current research in the field of bone metabolism remains largely confined to cellular and animal studies, with no clinical trials reported to date (Sun et al., 2024).

### 3.12 Dendrobium officinale polysaccharide

Dendrobium, known as one of the “Nine Immortal medicinal plant of China”, has the efficacy of “improving eyesight and tonifying the kidneys” and “nourishing yin and clearing heat.” Dendrobium polysaccharides mainly contain monosaccharides, such as mannose, glucose, xylose, and arabinose (Luo et al., 2024), and the contents of the former two are relatively high.

#### 3.12.1 DOP for osteoporosis

The Nrf2 protein binds to the antioxidant response element (ARE) in the promoter region of the cytoprotective gene, Kelch-like ECH-associated protein 1 (KEAP1), and is degraded by a ubiquitin protease, thus inhibiting intracellular Nrf2 levels. Wang Y. et al. (2023) found that Dendrobium officinale polysaccharides (DOP) effectively increased Nrf2 accumulation of Nrf2 in the nucleus; however, the level of Nrf2 mRNA did not change significantly, indicating that its mechanism of action was not related to mRNA transcription regulation. In subsequent studies, DOP was found to inhibit Nrf2 ubiquitination, causing Nrf2 accumulation of Nrf2 in Nrf2 nucleus. The detailed mechanism of action involves the interference of DOP with the interaction between KEAP1 and Nrf2, which promotes osteogenic differentiation.

Li et al. (2020) determined that DOP can inhibit RANKL-induced osteoclast differentiation, and by inhibiting NF-κB activity, it inhibits phosphorylation of p65, thus reducing subsequent expression of downstream related genes such as NFATc1. p38 is an important part of the MAPK pathway and plays an important role in inducing osteoclast differentiation; its phosphorylation is also inhibited by DOP. Therefore, DOP may play a role in inhibition of osteoclast formation. However, it is not yet clear whether DOP affects the remaining MAPK pathways.

#### 3.12.2 Shortcomings of DOP application

The pharmacological effects of DOP are broad, encompassing antioxidant, anti-inflammatory, anti-diabetic, and immunomodulatory activities. In the field of bone metabolism, while no direct research has been reported, its immunomodulatory and anti-inflammatory effects may offer potential benefits for bone health. Currently, DOP remain largely at the experimental research stage and have not yet been widely applied in clinical practice (He et al., 2022).

### 3.13 Morinda officinalis polysaccharide

Morinda officinalis is a valuable TCM and is known as one of the “four southern medicines”. It tonifies the kidney yang” and “strengthens muscles and bones”. Polysaccharides are active metabolites extracted from Morinda officinalis. Owing to different sources and extraction methods, the components of the Morinda officinalis polysaccharides (MOP) obtained vary. Some MOI exhibit anti-osteoporosis effects (Shen et al., 2020).

#### 3.13.1 MOP for osteoporosis

PGC-1α (Peroxisome proliferator-activated receptor gamma coactivator 1 alpha) is a protein crucial for regulating oxidative stress and plays a pivotal role in mitochondrial redox balance and maintaining ROS homeostasis (Abu et al., 2023). In a study by Rong et al. (2022), MOP treatment increased bone mineral density and relative levels of serum trace elements in ovariectomized rats. Its effect is related to inhibition of the PGC-1α/PPARγ pathway. The study showed that MOP can inhibit the PGC-1α/PPARγ pathway by detecting the protein levels of PGC-1α and PPARγ. The activation of the PGC-1α/PPARγ pathway counteracts the positive effects of MOP in the above-mentioned treatment of osteoporosis. After inhibiting the PGC-1α/PPARγ pathway of ovariectomized rats, the levels of superoxide dismutase (SOD), glutathione peroxidase (GSH-Px) and glutathione (glutathione, r-glutamyl cysteingl + glycine, GSH) in the serum decreased and the level of malondialdehyde (MDA) increased, suggesting that antioxidant capacity is enhanced and osteoporosis of ovariectomized rats is alleviated.

The G1 phase of the cell cycle is mainly involved in cell growth and preparation for the S phase. The S phase is the cell synthesis phase, which allows the cells to enter the proliferation stage. When a large number of cells remain in the G1 phase, DNA synthesis is blocked, thereby inhibiting mitosis and inducing apoptosis. Li et al. (2008) used MOP to reverse the effects of all-trans retinoic acid (RA)-induced cells to remain in the G1 phase and enter the S phase to a lesser extent, thus promoting osteoblast mitosis and inhibiting apoptosis. MOP also inhibited the decrease in Bcl-2 expression and the increase in Bax expression during osteoblast apoptosis and inhibited RA-induced osteoblast apoptosis.

MOP has also shown efficacy in the OPG/RANK/RANK signaling pathway (Jiao et al., 2023). In a study by Peng and Zhong (2020), regardless of the dose of MOP, the relative expression of OPG protein in all MOP groups was lower than in the sham operation group, significantly higher than in the castration group, and had a similar effect to the positive drug nilestriol (Fang, 2018).

#### 3.13.2 Shortcomings of MOP application

Morinda officinalis polysaccharides exhibit a variety of pharmacological effects, including anti-fatigue, immunomodulatory, anti-inflammatory, anti-oxidant, antidepressant, and neuroprotective activities. In the field of bone metabolism, Morinda officinalis polysaccharides can promote osteoblast proliferation and inhibit osteoclast activity, showing significant improvement in postmenopausal osteoporosis. Currently, research on Morinda officinalis polysaccharides remains largely confined to animal and *in vitro* studies and has not yet been widely applied in clinical practice (Yue et al., 2024).

## 4 Conclusions and future prospects

Pathogenic factors for osteoporosis are numerous and complex, encompassing genetic, pharmaceutical, disease-related, and environmental elements. These factors affect calcium absorption, increase calcium excretion, reduce re-absorption, activate osteoclast activity to increase bone resorption, inhibit osteoblast function, and reduce bone matrix and bone formation, thus affecting the balance of bone metabolism. When bone resorption exceeds bone formation for a prolonged time, osteoporosis occurs. Polysaccharides, excluding starch and cellulose, are the main active metabolites of Chinese herbal medicines. They usually act as bioactive substances to maintain or enhance the physiological functions of the body and have anti-diabetic, antiviral, anti-tumor, anti-inflammatory, antioxidative, anti-radiation, lipid-lowering, and immune regulatory effects.

This review mainly introduces polysaccharide metabolites in some traditional Chinese kidney tonifying medicines and their activities involving various different signaling pathways such as Wnt, PI3K/Akt/mTOR, GSK3β/β-catenin, BMP-2/Smads, OPG/RAK/RAKL, MAPK, ERS, NFATc1, and JAK2/STAT3, inhibiting or activating the signaling factors therein, thus inhibiting osteoclast activity or enhancing osteoblast activity, exerting an anti-osteoporosis effect. Additionally, several polysaccharide metabolites from others kidney-tonifying Chinese medicines also demonstrate beneficial effects on bone metabolism such as Cuscutae Semen polysaccharide promotes bone formation through BMP-2/Runx2/Smad5 signaling while inhibiting bone resorption via suppression of TRAP, NFATc1, and c-Fos. Cervi Cornus Colla polysaccharides exert anti-inflammatory effects by inhibiting IL-1 and IL-6 (Duan et al., 2023).

The extraction methods for polysaccharides metabolites include water extraction, alcohol precipitation, alkaline extraction, ultrasound-assisted extraction, microwave-assisted extraction, enzymatic extraction, microbial fermentation, and supercritical CO_2_ extraction. Water extraction is widely used but generally yields low efficiency, while alcohol precipitation is often paired with water extraction yet offers limited yields. Alkaline extraction is common but can affect polysaccharide structures. Ultrasound and microwave-assisted extractions improve efficiency but may involve higher equipment costs. Notably, for APS, novel techniques like subcritical water-assisted extraction and flash extraction are emerging to enhance efficiency, while microbial fermentation maintains structural integrity but presents quality control challenges (Tian et al., 2024; Ba et al., 2025). For LBP, dual-frequency ultrasound extraction can increase yields by 73.41% compared to traditional methods (Wang et al., 2024; Zhou et al., 2020). Composite extraction methods for PSP achieve yields over 30%, albeit with increased complexity (Guo et al., 2025). Lastly, microwave-assisted extraction stands out for ABP due to its high efficiency (Chai et al., 2024), while ultrasound-assisted and microwave-assisted techniques are particularly effective for polysaccharides from Rehmannia glutinosa and Morinda officinalis (Jia et al., 2023; Bian et al., 2023; Yue et al., 2024).

Polysaccharides from TCM hold great promise for the prevention and treatment of osteoporosis. Additionally, several polysaccharide metabolites from kidney-tonifying Chinese medicines demonstrate beneficial effects on bone metabolism. Cuscutae Semen polysaccharide promotes bone formation through BMP-2/Runx2/Smad5 signaling while inhibiting bone resorption via suppression of TRAP, NFATc1, and c-Fos. Dipsaci Radix polysaccharide improves bone microcirculation through PI3K/Akt/eNOS-mediated VEGF upregulation and modulates the RANKL/OPG axis. Euodiae Fructus polysaccharides (MOP70-2 and MOW90-1) stimulate osteoblastic differentiation by upregulating Runx2, osterix, and osteoprotegerin. Cistanches Herba polysaccharide activates Wnt/β-catenin signaling to enhance osteogenesis. Eucommiae Cortex polysaccharide enhances immune function by increasing thymus and spleen coefficients and macrophage phagocytosis. Cervi Cornus Colla polysaccharides exert anti-inflammatory effects by inhibiting IL-1 and IL-6.

These polysaccharides coordinately regulate bone remodeling through multiple molecular pathways. However, several challenges must be addressed before these polysaccharides can be effectively applied in osteoporosis therapy. For instance, the extraction rate of refined polysaccharides from TCMs is relatively low, most polysaccharide extracts are crude polysaccharides, the technology to extract a large amount of highly active refined polysaccharides, clarifying the pharmacodynamic structure of polysaccharides and a detailed understanding of the mechanism of action of polysaccharides. Polysaccharides from TCM demonstrate poor oral bioavailability yet substantial pharmacological activity, attributed to their unique PK-PD relationship. Predominantly unabsorbed TCMPs elicit local intestinal effects-anti-inflammation, microbiota modulation, and immune regulation that translate into systemic benefits through gut-organ axes. Limited absorption occurs via lymphatic routes and receptor-mediated mechanisms, yielding selective hepatic, renal, and tumoral distribution for direct tissue protection. This PK profile, defined by intestinal retention rather than systemic exposure, constitutes the mechanistic basis for TCMP efficacy (Ye et al., 2023). However, there are few studies on the pharmacokinetics of traditional Chinese medicine polysaccharides in the field of bone metabolic diseases. Preclinical studies in animal models and cell cultures demonstrate promising results for Polysaccharides from TCM the majority of these findings have not been validated in human clinical trials. Future research should prioritize rigorous clinical studies to confirm efficacy and safety in human populations. These represent the current major challenges in the field. Further research and development of polysaccharide metabolites of TCM in medicine can provide new incentive for the development and improvement of drugs for the prevention and treatment of osteoporosis.

## Glossary

Traditional Chinese medicine (TCM): Traditional Chinese medicine is a general term for traditional Chinese medicine including Han nationality medicine and ethnic minority medicine. Traditional Chinese medicine has a long history, and its origin can be traced back thousands of years ago. The holistic concept in the basic theory of traditional Chinese medicine believes that the human body is an organic whole, and various organs and tissues interact with each other physiologically and pathologically.
Tonification of the kidney: Tonification of the kidney is a traditional Chinese medicine treatment method mainly for the situation of insufficient or weak kidney function. The kidney in traditional Chinese medicine is a functional system and is not equivalent to the kidney in modern medicine. Whether the function of the kidney in traditional Chinese medicine is normal has a crucial impact on human growth and development, reproduction, fluid metabolism, respiration and other aspects.
